# What’s New with Flu? An Overview

**DOI:** 10.3390/v11050433

**Published:** 2019-05-10

**Authors:** Seema S. Lakdawala, Christopher B. Brooke

**Affiliations:** 1 Department of Microbiology & Molecular Genetics, University of Pittsburgh School of Medicine, Pittsburgh, PA 15219, USA; 2 Center for Vaccine Research, University of Pittsburgh School of Medicine, Pittsburgh, PA 15219, USA; 3 Department of Microbiology, University of Illinois at Urbana-Champaign, Urbana, IL 61801, USA; 4 Carl R. Woese Institute for Genomic Biology, University of Illinois at Urbana-Champaign, Urbana, IL 61801, USA

One hundred years have passed since the 1918 H1N1 pandemic, and influenza viruses continue to pose an enormous and unpredictable global public health threat. Recent technological and methodological advances have greatly expanded our understanding of the basic biology of influenza infection, shedding new light on old problems in the influenza field. This special issue focuses on recent discoveries, primarily by young investigators, that have emerged from the use of novel approaches to study long-standing questions in influenza biology. 

This special edition contains 7 reviews, 9 primary research articles, 1 opinion, and 1 educational article. Of these, 29% of the reviews are from labs of assistant professors. We are pleased that 57% of the reviews contain either female lead authors and/or female corresponding authors. Primary research articles contain 33% female lead authors and/or female senior authors. This includes an opinion piece by Dr. Kanta Subbarao, Director of the WHO Collaborating Centre for Reference and Research on Influenza in Australia, describing the top ten advances in influenza virus research in the past few decades [1].

In addition to the research and review articles, this issue includes an article containing educational material for students from kindergarten to Grade 12 related to a selection of articles included in this special edition. This article includes simple handouts with fun activities regarding different aspects of influenza virus biology and a fact sheet about influenza virus infections [2]. The goal of including these materials is to increase the awareness of and the accessibility to both ongoing influenza research as well as key information about influenza virus infection among the lay public.

A particular focus of this special edition is on the use of new techniques and approaches to examine key questions in influenza biology. Specifically, multiple papers highlight the exciting potential of mathematical modeling to shed new light on a variety of topics, including viral evolution from vaccination [3], the spatial structure of within-host infection [4], and the effects of neuraminidase inhibitors [5]. It is becoming increasingly clear that the combination of experimental and modeling approaches has the potential to greatly expand our understanding of infection biology.

Another important hot topic covered by several articles in this special edition is the role of viral and host RNA during infection. These include the identification of a novel long non-coding RNA that regulates viral replication [6], a characterization of the NP-viral RNA binding profiles for H3N2 and influenza B viruses [7], and a review of viral mechanisms of host shut-off [8]. As RNA sequencing and RNA biology technologies continue to develop, we are certain to enjoy many new discoveries surrounding the intricacies of host and viral RNA regulation during infection.

Another critical aspect of influenza biology explored in this special issue is the relationship between the glycoproteins (HA and NA) and the ability to target them with vaccine strategies. In this special edition, Drs. Ivan Kosik and Jon Yewdell have written a comprehensive review on the intimate functional relationship between HA and NA during viral infection [9]. Reviews covering live attenuated vaccine platforms [10] and the pandemic threat of emerging avian influenza viruses [11] provide insight into these important public health areas.

Finally, a number of articles cover new fronts in our efforts to understand the evolution of influenza viruses. Lyons and Lauring review what is known about the effects of mutation and epistasis during influenza evolution [12]. Slaine et al. detail a study aimed at understanding the role of polymerase mutations during host adaptation [13]. In addition, many of the articles already mentioned explicitly cover specific aspects of influenza evolution that have been relatively understudied to date, including functional constraints between the glycoprotein genes, and the spatial structure of viral populations within hosts [4,9].

We were thrilled to edit this special edition and bring together so many wonderful new approaches to all aspects of influenza research. There is a lot “new in Flu” and we are excited to see where the field goes over the next several years.
